# Mental health of doctors in a tertiary hospital in Nigeria

**DOI:** 10.11604/pamj.2014.19.178.3642

**Published:** 2014-10-21

**Authors:** Baba Awoye Issa, Abdullah Dasliva Yussuf, Ganiyu Toyin Olanrewaju, Olatunji Alao Abiodun

**Affiliations:** 1 Department of Behavioural Sciences, College of Health Sciences, University of Ilorin, Nigeria

**Keywords:** Doctors, psychiatric morbidity, tertiary hospital, Nigeria

## Abstract

**Introduction:**

Doctors are vulnerable to psychiatric morbidity as a result of their busy schedules and multiple role obligations. Yet, they often don't admit they have mental health problems nor are they readily subjected to mental health evaluation by their colleagues due to fear of labeling and general stigma.

**Methods:**

A cross-sectional survey of doctors in the service of University of Ilorin Teaching Hospital, Ilorin, Nigeria was done using a socio-demographic questionnaire and the twelve items General Health Questionnaire (GHQ-12) using a cut-off point of 3 to indicate possibility of psychiatric disorder (GHQ-12 positive). Non-parametric analysis and regression test of factors associated with psychiatric morbidity was done using SPSS. Level of significance was set at 0.05 p-value.

**Results:**

Two hundred and forty one doctors representing 68.9% of the doctors participated in the survey. The point prevalence of psychiatric morbidity among the doctors using the GHQ-12 was 14.9%. Being married, non-participation in social activities and perception of work load as being “heavy” were significantly associated with psychiatric morbidity (p-value < 0.05).

**Conclusion:**

The prevalence of psychiatric morbidity among doctors at the University of Ilorin Teaching Hospital was higher than the general population prevalence. Measures to lessen the negative effect of marriage and the perceived heavy work load on mental health of doctors, such as provision of recreational facilities within the hospital and encouragement of doctors’ participation in social activities are advanced.

## Introduction

Medical doctors are known to take care of patients. However in the process of doing this primary assignment they often pass through some stresses related to their occupation. Doctors have also been reported to neglect their own health [1]. Despite the work stress, doctors find it difficult to admit that their work is stressful, that they have abnormal coping strategies such as drinking or drug-related problems or even to express that they need help for these problems [2]. A Nigerian study has corroborated some of these submissions in a study that reported that 4.1% of the study population of doctors were hazardous users while 12.0% were moderate users of alcohol [3]. Therefore, doctors seem to have higher rates of mental disorders and do not report these problems early enough as a result of stigma, the culture of “always coping”, fear about damaging job prospect, and uncertainty about who to tell [2, 4].

Aside drinking or drug-related problems, suicide rates, though under-reported, also appeared high in female doctors, Anaesthetists, General Practitioners (GPs) and Psychiatrists [5, 6]. Arguably, doctors’ access to prescription drugs could play a part in their risk of substance use and suicide, as well as making it easier to treat themselves rather than seeking help. Job stress, burnout and mental ill health could lead to medical errors, poor judgement, poor interpersonal and inter- professional relationships and poor quality care [7–10].

The implication of these is that doctors, like their patients, also need periodic mental health evaluation and treatment when mental ill health is detected. However, just as mentally sick doctors have difficulty disclosing their problems with others, other doctors are also uncertain of initiating assessment of fellow doctors for mental ill health [11]. The fear of labelling due to general suspicion and prejudice about psychiatric disorder within society and counter accusation for character defamation might possibly be reasons for low level of mental health assessment of colleague doctors [11]. This has once necessitated why a study posed a question that “if we cannot overcome our own reluctance to face and accept the reality of psychological illness, what hope is there for the rest of the society?” [11].

Reported main sources of stress among doctors include work pressure, high job demand, poor relationships with colleagues, particularly poor team working and service pressures, domestic stressors such as taking care of small children and competing tensions of work and home life, isolation, lack of peer support and marginalisation particularly for women and minority groups [12]. In addition, they are not only responsible for assessment, diagnosis, and treatment of their patients (or clients), they are also involved in all manners of related administrative and practice responsibilities. It's no wonder then that they are too busy to step back and periodically assess themselves and their practices.

This study aimed at determining the mental health of doctors in a Nigerian University Teaching Hospital, thus serving as a step forward in self-assessment of mental health of doctors by colleague doctors. Findings from this study might be useful in planning for better mental health care for our doctors in order to enable them treat the wider population of Nigerians more appropriately.

## Methods

The data for this study are part of a larger study to determine the doctors’ management skill of the mentally ill, psychological impact of work on the doctors as well as their alcohol use [3]. It was done at the University of Ilorin Teaching Hospital (UITH), a tertiary health centre that provides health services for Kwara State, one of the 36 States in Nigeria and the surrounding states. Enveloped questionnaires together with information sheets and consent forms were distributed to all doctors in the service of the hospital irrespective of their cadre in all the departments except the department of Behavioural sciences, the department of the researchers. The questionnaires included a socio-demographic questionnaire detailing age, gender, marital status, professional qualification; clinical parameters such as history and nature of prior treatment for any form of emotional disturbances by the participants or their relatives; work related conditions such as leisure, social club participation, perceived cordiality with co-workers and patients, perception of workload and remuneration satisfaction. They also completed 12-items General Health Questionnaire (GHQ-12) which was used to assess their emotional dysfunction. A score of 3 or more on the GHQ-12 was regarded as positive, indicating the possibility of psychiatric morbidity [13, 14]. The Ethics and Research Committee of the hospital approved the study protocol.

### Statistical analysis

Descriptive statistics were recorded for the questions and univariate comparisons were performed using chi-square. Statistical significance was set at the 5% level.

## Results

Of the 350 respondents who were served with questionnaires, 241 returned filled and analysable questionnaires, thus giving a response rate of 68.9%. Fifteen (6.2%) had sought treatment for emotional disturbances in the past, majority (153 or 63.5%) preferred relaxation with family members as leisure, few (11 or 4.6%) preferred engagement in religious activities, and 4 (1.7%) did not indicate their leisure preferences. Most (195 or 80.9%) of the cohort perceived their workload as “satisfying” compared to 186 (77.2%) who perceived their job as “heavy”. Thirty-four (14.9%) respondents scored ≥ 3 on the GHQ-12 implying having psychiatric morbidity (Table 1). Thus the point prevalence of psychiatric morbidity among the doctors was 14.9%. Marital status, participation in social activities, perception of work load, and satisfaction with work were significantly associated with psychiatric morbidity (p-value < 0.05, Table 2, Table 3). Gender, professional qualification, practice group, remuneration, and previous seeking or treatments for emotional disturbance were not statistically related to development of psychiatric morbidity.

**Table 1:** Socio-demographic characteristics and GHQ-12 scores of respondents

GHQ REPORT			
Variables	GHQ –VE	GHQ +VE	p-value
**Gender**			0.57
Male	155	27	
Female	52	7	
**Marital status**			0.00
Single	26	13	
Married	181	21	
*Professional qualification			0.94
MB;BS only	79	14	
Primary	37	4	
Part I	44	7	
Part II	47	9	
**Practice group**			0.19
Surgeons	96	20	
Physicians (internists)	104	11	
Others (Gen Pract/Med Off)	7	3	

* Primary, Part I and Part II refer to the stages (beginner, intermediate and final) of examinations passed at the Postgraduate Medical Colleges in Nigeria

**Table 2:** Description of work/leisure and Ghq-12 Scores of the respondents

	GHQ-12 Negative	GHQ-12 Positive	p-value
**Assessment of work load**			0.00
Heavy	152	34	
Not heavy	55	0	
**Work satisfaction**			0.00
Satisfying	174	21	
Not satisfying	33	13	
**Leisure:**			0.84
Relaxing with family	135	18	
Travelling	46	11	
Playing games	13	3	
Religion activities	9	2	
Not indicated	4	0	
**Participation in Social activities**			0.00
Very often (weekly)	30	0	
Often (forth nightly)	26	4	
Occasionally (monthly)	87	3	
Not at all	62	27	
**Assessment of remuneration**			0.91
Very commensurate	2	0	
Commensurate	72	13	
Not commensurate	129	21	
**Ever sought/received treatment for emotional disturbances**			0.22
Yes	15	0	
No	192	34	

**Table 3:** Logistic regression

Variables	B	S.E.	Wald	df	Sig.	Exp (B)
Marital status	-1.377	.478	8.289	1	.004	.252
Participation in social activities	1.173	.364	10.398	1	.001	3.233
Perception of work load	-.922	.310	8.825	1	.003	.398
Satisfaction with work	.725	.366	3.926	1	.048	2.065

## Discussion

### Psychiatric morbidity among doctors

A prevalence of 14.9% psychiatric morbidity among doctors in this study was slightly higher than the Nigerian general population prevalence of 12.1% [15]. In the United Kingdom, the prevalence of psychiatric morbidity among doctors in the NHS was 18.1% compared with l7.8% of people in the general population [16]. Grassi and Magnani reported morbidity levels of 20.3% and 24.6% among Italian general practitioners and hospital physicians respectively [17]. It however, compared fairly well with a prevalence of 14.2% among Nigerian Executives [18] but lower than 18.9% prevalence among hospital consultants in the same hospital few years earlier [19]. This result thus agree with studies from some other parts of the world of poorer mental status than the general population.

### Factors associated with psychiatric morbidity

After a logistic regression, factors associated with significant morbidity included the marital status, the work load, work satisfaction, and participation in social activities Table 3. With almost two-third of doctors with psychiatric morbidity being married, marriage thus appeared to be a factor in the development of psychological morbidity. This seems to contradict the earlier believe that marriage is a protective factor for psychological disorders [20]. A plausible reason is the additional role marriage places on a busy doctor, particularly female doctors who would have to take care of the husband and children in addition to doing her work as a physician [11]. Gender was not statistically significant in this study, although more men than women appeared to have psychiatric morbidity. Therefore, if marriage was an albatross for male doctors, further researches might be necessary to unravel this, but possible factors such as financial and moral responsibilities that are naturally imposed on men might be responsible. Perception of work load as “heavy ” and non-participation in social activities were significant factors in having a psychiatric morbidity. This study agrees with the finding by McManus [21] that job stress was associated with emotional exhaustion and psychiatric morbidity. It also supports the interactional model of stress that stress at work resulted in poor mental health [22, 23].

Above factor (perception of work load) is linked with satisfaction with work. In this study, however, despite the fact that all the respondents perceived their work load as ”heavy“ majority were satisfied with the work. This finding might be due to the fact that the doctors were using the job contentment as a defence mechanism against stress and psychological breakdown. This reasoning reckons with that of McManus that job satisfaction protects an individual from psychological morbidity by reducing the likelihood of developing emotional exhaustion, and was associated with higher personal accomplishment [21]. The mental health benefit of participation in organized physical recreation and social activities has been documented. People who participate in sports, clubs and organized recreational activity enjoy better mental health, are more alert, and more resistant to the stresses of modern day living [24]. This study confirms this because those who indicated not participation in social activities were mostly those who had psychiatric morbidity.

## Conclusion

The prevalence of psychiatric morbidity among doctors at the University of Ilorin teaching Hospital was 14.9%. This figure is higher than the general population prevalence. Being married, perception of work load as ”heavy“ and non-participation in social activities negatively impacted on the mental health of doctors. To stem this tide there should be measures to lessen the negative effect of marriage on doctors. This could be in areas of reducing the distance between home and work by building staff residences within the hospital or close-by and encouraging the doctors to use them. Another step could be in establishment of baby care centres/crèche and elementary staff school within the hospital and for doctors to employ domestic assistants to help with homework. Other services could include regular meals for doctors’ on-call. Doing these perhaps would reduce the negative impacts of the perceived heavy work load. Provision of recreational facilities within the hospital and encouraging doctors to participate in social activities might greatly help reduce psychiatric morbidity among doctors.
